# Analysis of Incidence and Risk Factors of Complicated Acute Appendicitis in Children: Evidence From a Tertiary Pediatric Center

**DOI:** 10.1155/ijpe/1230753

**Published:** 2025-03-23

**Authors:** Sina Azadnajafabad, Faraha Zahra Awan, Bahar Ashjaei, Hosein Alimadadi, Mahsa Soti Khiabani

**Affiliations:** ^1^Children's Medical Center, Pediatrics Center of Excellence, Tehran, Iran; ^2^Department of Surgery, Tehran University of Medical Sciences, Tehran, Iran; ^3^Non-Communicable Diseases Research Center, Endocrinology and Metabolism Population Sciences Institute, Tehran University of Medical Sciences, Tehran, Iran; ^4^Tehran University of Medical Sciences, Tehran, Iran; ^5^Department of Pediatrics, Tehran University of Medical Sciences, Tehran, Iran; ^6^Pediatric Gastroenterology and Hepatology Research Center, Tehran University of Medical Sciences, Tehran, Iran

**Keywords:** appendicitis, complications, pediatrics, risk factors, incidence, Iran

## Abstract

**Background:** Acute appendicitis (AA) is the most prevalent surgical emergency in the pediatric population, with the complicated form leading to various adverse outcomes. Our study is aimed at evaluating the incidence and associated risk factors of complicated AA among children presenting with this condition.

**Methods:** Employing a cross-sectional design, we included all children suspected of having AA who were admitted to a tertiary pediatric center in Iran from 2020 to 2021. Pathologists examined all surgically removed appendices, and only cases with histopathological confirmation of AA were included. We classified AA into complicated and uncomplicated categories. We recorded and analyzed demographic, clinical, and laboratory data of patients admitted with AA. Analyzed laboratory parameters included white blood cell (WBC) count, neutrophil count and percentage, erythrocyte sedimentation rate (ESR), and C-reactive protein (CRP).

**Results:** The study comprised 98 pediatric patients with AA, including 60 males (61.2%) and 38 females (38.8%), with a median age of 9.0 (interquartile range: 7.0–11.0) years. Eighteen (18.4%) cases were diagnosed with complicated AA. Mean WBC count, neutrophil count, and CRP levels were significantly higher in patients with complicated AA (*p* values: 0.048, 0.018, and 0.014, respectively). After adjusting for relevant clinical factors, CRP (odds ratio: 1.02 [95% CI: 1.00–1.04]), WBC count (1.18 [1.03–1.37]), and neutrophil count (1.23 [1.06–1.45)]) were significantly associated with complicated AA. Receiver operating characteristic (ROC) curve analysis indicated a CRP cut-off of 19.5 mg/dL, with an area under the curve of 0.687 (95% CI: 0.551–0.823), a sensitivity of 72.2%, and a specificity of 68.4% for predicting complicated AA.

**Conclusions:** Laboratory parameters, specifically WBC count, neutrophil count, and CRP levels, are significant independent predictors of complicated AA in pediatric patients. These findings could assist in the timely diagnosis and management of children suspected of having AA in clinical practice.

## 1. Introduction

Acute appendicitis (AA) represents one of the most common nontraumatic surgical emergencies in the pediatric population [1]. Recent estimates from 2019 indicate that appendicitis in individuals under 20 years old has a global incidence rate of approximately 271 new cases per 100,000, exhibiting a slight increase over the past three decades [2]. The significant prevalence of this pediatric surgical condition translates into substantial healthcare service utilization and associated costs [3]. Despite diagnostic advancements, the atypical presentation of AA in children, coupled with communication challenges in early childhood and distinct anatomical and pathophysiological differences, complicates diagnosis, often resulting in delays [4, 5].

Complicated AA, characterized by a perforated or gangrenous appendix, intraabdominal abscess/phlegmon, or severe peritonitis, is linked to increased morbidity and mortality [6, 7]. Approximately 35% of pediatric AA cases are complicated, underscoring the need for prompt diagnosis and effective management [8]. Ongoing research has identified several risk factors and biomarkers associated with complicated AA in children, such as delayed presentation, symptom duration exceeding 24 h, age under 5 years, the presence of an appendicolith, elevated leukocyte counts, hyponatremia, increased procalcitonin levels, and high C-reactive protein (CRP) levels [6, 7, 9, 10].

Given the prevalence and potential severity of complicated AA in children, numerous studies have developed prediction models incorporating clinical, laboratory, and imaging factors to assess patient prognosis and distinguish complicated cases, aiming to improve outcomes [10–16]. Ultrasound (US), commonly used in emergency departments to detect AA, has relatively poor predictive value in children suspected of having AA, and due to these variations in pediatric patients, a robust consensus on definitions and diagnostic criteria for AA in children is still lacking [17]. While combining results from different diagnostic modalities can aid in identifying complicated AA, there is an increasing interest in using simple laboratory markers to diagnose severe cases promptly, preparing patients for surgery, and averting the adverse outcomes of a perforated or gangrenous appendix [10, 16, 18].

In this study, we aim to investigate both complicated and uncomplicated pediatric AA cases in a referral tertiary pediatric center in Iran. By assessing clinical and laboratory factors, our goal is to provide insights that will assist clinicians in effectively managing complicated pediatric AA cases, particularly in developing countries with limited healthcare resources.

## 2. Materials and Methods

### 2.1. Study Design and Population

We conducted this study using a retrospective cross-sectional design at the Children's Medical Center, a tertiary pediatric referral center affiliated with Tehran University of Medical Sciences in Tehran, Iran, during the 2020–2021 period. Deidentified medical records were accessed on July 10, 2023, for data collection and analysis. In this process, the authors had no access to information that could identify individual participants during or after data collection. We employed a simple convenience sampling method to enroll patients. Only children suspected of having AA and admitted to the center were considered. Postsurgery, a pathologist examined all surgically removed appendix tissues, and cases with histopathological confirmation of AA were included in the study. Exclusion criteria encompassed patients with AA ruled out during surgery, those with non-AA pathology reports postsurgery, and patients with congenital or acquired immunodeficiency.

### 2.2. Definition of Variables and Data Collection

We recorded demographic, clinical, and laboratory data of patients diagnosed with AA. Patients were categorized into complicated and uncomplicated groups. Complicated AA included cases with a perforated or gangrenous appendix, abscess formation, or peritonitis. These classifications were confirmed both clinically and paraclinically by the pediatric surgeon and pathologist pre and postsurgery. Demographic variables included age and sex. Clinical variables assessed were symptom duration from disease onset, evidence of AA and appendix diameter on US, fever, diarrhea, and abdominal tenderness. Laboratory parameters for analysis encompassed white blood cell (WBC) count (∗10^9^/*L*), neutrophil count (∗10^9^/*L*) and percentage, erythrocyte sedimentation rate (ESR) (millimeters per hour), and CRP (milligrams per deciliter). WBC and neutrophil counts were further categorized into high (≥ 10000 and ≥ 7500, respectively) and low (< 10000 and < 7500, respectively) groups.

### 2.3. Statistical Analysis

The normality of quantitative variables was tested by the Shapiro–Wilk normality test and reported the normally distributed variables in mean ± standard deviation (± SD), and the nonnormal distributed variables in the median with first quartile and third quartile (Q1–Q3). Qualitative variables were reported as frequencies and percentages. We used the chi-squared or Fisher's exact test for categorical variables and the independent samples *t*-test or Mann–Whitney *U* Test for quantitative variables, based on data normality. Variables significantly associated with complicated AA were further examined using receiver operating characteristic (ROC) curve analysis to determine predictive cut-offs. Sensitivity, specificity, and area under the curve (AUC) results were reported. The Youden index identified the most effective cut-off in the ROC analysis. Univariable logistic regression analysis, followed by multivariable logistic regression using the enter method and adjusted for clinically relevant factors, identified laboratory markers independently associated with complicated AA. The Hosmer–Lemeshow test evaluated the logistic regression models' fit, reporting odds ratios (ORs) with 95% confidence intervals (CI). A two-tailed *p* value < 0.05 was considered statistically significant. Data curation, analysis, and visualization were done using the R programming language, Version 4.4.2 (R Foundation for Statistical Computing, Vienna, Austria, https://www.R-project.org/).

### 2.4. Ethical Considerations

The study protocol received ethical approval from the committee at Tehran University of Medical Sciences (code: IR.TUMS.CHMC.REC.1401.147). Parents or guardians of participating patients provided informed consent, and they were free to withdraw from the study at any stage. This study was in accordance with the ethical standards of the Tehran University of Medical Sciences and with the World Medical Association's 1964 Declaration of Helsinki and its later amendments.

## 3. Results

In our study, we included 98 pediatric patients with AA, consisting of 60 males (61.2%) and 38 females (38.8%). The median age was 9.0 (interquartile range: 7.0–11.0) years. Among these, 18 (18.4%) cases were identified as complicated AA, while 80 (81.6%) were uncomplicated. Notably, all nine patients (9.2%) under 5 years old presented with uncomplicated AA. US examination revealed evidence of AA in 88 patients (89.8%), while 10 cases (10.2%) showed no specific signs of AA before surgery. The median appendix diameter for the sample, excluding the nine patients with a normal appendix diameter, was 0.8 cm (0.5–1.0). Over half of the patients experienced symptoms for less than a day, a pattern observed in both complicated and uncomplicated groups. Diarrhea as a symptom was reported in 16.3% of cases. Right lower quadrant (RLQ) tenderness was the most common clinical finding (39.8%), with a similar distribution across both groups. Leukocytosis was present in 76 cases (77.6%), while 22 (22.4%) had normal WBC counts. Elevated neutrophil counts were observed in 68 patients (69.4%), with the remaining 30 (30.6%) showing normal values.

Demographic and clinical symptoms did not differ significantly between complicated and uncomplicated AA cases. Laboratory analysis revealed that while ESR was higher in complicated AA cases with a median of 23.0 (14.0–51.0) compared to uncomplicated ones with a median of 15.0 (8.0–26.0), this difference was not statistically significant (*p* value = 0.075). Additionally, CRP levels were significantly higher in complicated AA cases with a median of 34.0 (8.0–58.0) than in uncomplicated cases with a median of 8.0 (2.0–35.0), with a *p* value of 0.014. A similar significant difference was observed in the mean WBC count, with higher levels in complicated cases with a median of 16.7 (11.6–21.2) (∗10^9^/*L*) compared to uncomplicated ones with a median of 12.6 (9.9–15.8) (∗10^9^/*L*), yielding a *p* value of 0.048. This significant trend continued for median neutrophil count (14.4 in complicated vs. 9.5 (∗10^9^/*L*) in uncomplicated cases, *p* value =0.018), but not for neutrophil percentage (Table 1).

Unadjusted regression analysis indicated that higher values of ESR (OR: 1.03 [95% CI: 1.00–1.05]), WBC count (OR: 1.10 [1.00–1.21]), and neutrophil count (OR: 1.12 [1.01–1.24]) were significantly associated with complicated AA in children. Following adjustment for relevant clinical factors, all four laboratory factors, including ESR (1.10 [1.04–1.18]), CRP (1.02 [1.00–1.04]), WBC count (1.18 [1.03–1.37]), and neutrophil count (1.23 [1.06–1.45]), were significantly and independently associated with complicated AA (Table 2).

**Figure 1:**
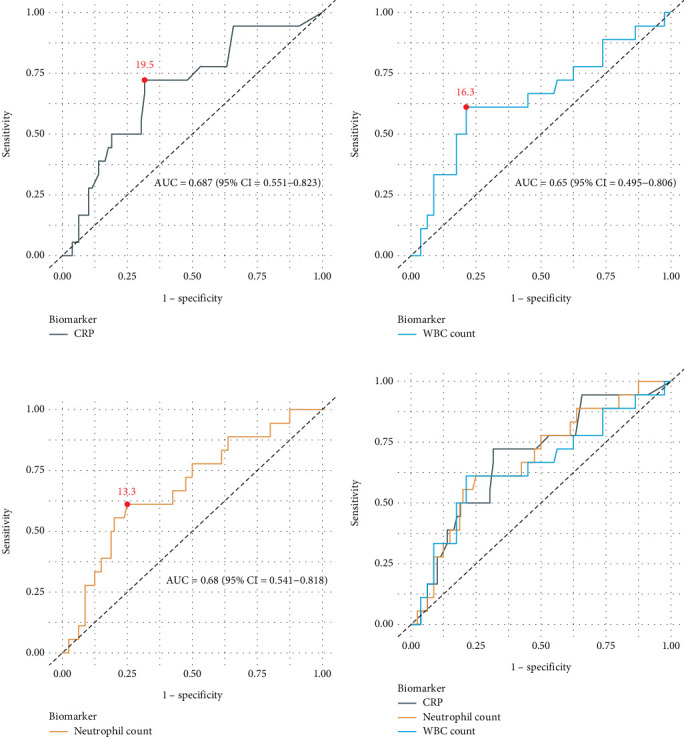
ROC curve analysis of significant factors associated with complicated acute appendicitis in children, (a) C-reactive protein, (b) white blood cell count, (c) neutrophil count, and (d) all three markers in one model.

ROC curve analysis of CRP, WBC count, and neutrophil count, which showed significant associations with complicated AA, revealed a CRP cut-off of 19.5 with an AUC of 0.687 (95% CI: 0.551–0.823), a sensitivity of 72.2%, and a specificity of 68.4%, predictive of complicated AA at or above this threshold (*p* value = 0.013). For WBC count, a cut-off of 16.3 (∗10^9^/*L*) with an AUC of 0.65 (0.495–0.806), a sensitivity of 61.1%, and a specificity of 78.8% was predictive of complicated AA (*p* value = 0.047). The neutrophil count cut-off of 13.3 (∗10^9^/*L*) showed an AUC of 0.68 (0.541–0.818), a sensitivity of 61.1%, and a specificity of 75.0%, indicating a higher likelihood of complicated AA (*p* value = 0.017). When incorporating all three factors into the ROC curve model simultaneously, only CRP emerged as the best predictor of complicated AA (Figure 1).

## 4. Discussion

This study examined the incidence of complicated AA in pediatric patients and assessed various clinical and laboratory factors associated with complicated AA cases. Our key findings indicate that approximately one-fifth of AA cases were complicated, with elevated levels of CRP, WBC count, and neutrophils being significantly associated with complicated AA. These markers could serve as practical paraclinical tools for predicting complicated AA in pediatric patients.

We found that 18.4% of our AA cases were complicated, a rate consistent with similar studies in pediatric populations. For example, rates of complicated AA have been reported as 42.9% and 32.3% in China [18, 19], 14.3% in Ethiopia [20], 32.5% and 35.1% in United States [8, 21], 48.6% and 20.4% (in younger children < 10 years old) in Korea [12, 14], 27.3% in India [7], and 16.8% in Sweden [22], with significant variation observed across different countries and healthcare settings. These differences highlight the importance of understanding local healthcare dynamics and patient characteristics when interpreting such data.

In our study, about 10% of patients were under 5 years old, and none had complicated AA. However, other studies show varying rates for this age group. For instance, one study reported 17% complicated cases in children under 5 [10], another found rates of 46% and 19% for gross perforation and abscess, respectively [21], and a study in China showed a 63.9% complication rate in this age group [13]. Another study reported the share of complicated AA cases in < 5 years old group as 44.4% which was higher than the 5–10 years (12.5%) and 10–14 years (17.0%) age groups [23]. These inconsistencies in the prevalence of complicated AA in younger children highlight the need for further research to establish more consistent patterns.

As reviewed above, the proportion of complicated AA cases in pediatric patients varies widely, and the difference could be due to different study designs, statistical methods, and variations in population characteristics around the world [1, 2]. The statistics of the current investigation were lower compared to most of those studies, and this could be attributed to several reasons. One justification could be Iran's accessible and affordable primary healthcare system, which promptly refers patients needing surgery to the secondary and tertiary referral centers and this might contribute to the lower incidence of complicated AA in Iran [24, 25]. Another justification could be the possibility and accessibility of making direct visits at a tertiary pediatric center even without a referral from lower medical centers, which might help in timely diagnosis, reducing the incidence of complicated AA cases in Iran [26].

Timely diagnosis of AA in children and appropriate treatment in eligible cases is a highly important issue to prevent adverse outcomes. Perforated cases are associated with more morbidity (higher readmission, postoperative intra-abdominal abscess, and surgical site infection rates) compared to simple cases [4, 17]. Also, septic shock as an important risk factor of morbidity and mortality often occurs in complicated appendicitis cases [27]. Therefore, a right knowledge of the risk factors of complicated AA in children and beneficial biomarkers would help clinicians in the management of these cases [3, 28].

One of the main findings of this study was that CRP, WBC, and neutrophil count are predictors of complicated pediatric AA, each with acceptable sensitivity and specificity. These results are consistent with similar studies. For example, one study reported that a WBC count > 13,500 and a neutrophil percentage > 62% were significantly associated with perforated AA [20]. Another found that a leukocyte count >15∗10^9^/*L* was present in about 50% of complicated AA cases, compared to 15.6% in uncomplicated cases [7]. A comparable study reported that WBC count, CRP, and neutrophil count were significantly higher among perforated AA; however, in the adjusted analysis, only the combination of WBC + CRP and neutrophils + CRP were significant predictors of complicated AA [19].

One analysis of pediatric AA showed that CRP was significantly higher in complicated cases, predicting them with a cut-off of 4.0 mg/dL (sensitivity: 69%; specificity: 83%) [29]. Another study found CRP and WBC, but not neutrophils, significantly higher in complicated cases, with CRP > 40 mg/L having a positive predictive value of 38% and negative predictive value of 80% for complicated AA [16]. In a study on children under five, WBC was the only significant marker [13]. Additionally, *WBC* > 14.7∗10^9^/*L* showed 63.3% sensitivity and 69% specificity, and CRP > 7.1 mg/dL showed 50% sensitivity and 83.7% specificity for the detection of complicated AA [30]. One study found *CRP* ≥ 50 *mg*/*L* as a significant marker for complicated AA [14]. In cases of septic shock following AA in children, CRP and procalcitonin levels were notably elevated [27].

In this investigation, we found no differences in age, sex, signs, or symptoms between complicated and uncomplicated AA cases. However, other studies report varied findings. Some studies identified younger age, longer symptom duration, and generalized abdominal tenderness as significant risk factors for complicated AA [7, 20, 31]. For example, symptom duration > 24 h was often associated with more severe cases [6, 20]. Consistent with our findings, some studies showed similar patterns of fever, diarrhea, and RLQ tenderness between groups [19, 29]. Differences in clinical presentations across studies may result from varying definitions and measurements of signs and symptoms, highlighting the need for careful interpretation and comparison.

The markers identified in this study, along with those from similar publications, can help clinicians diagnose and manage pediatric AA cases, particularly complicated ones, to prevent adverse outcomes. However, these measures should be used with caution and tailored to each patient's condition. Importantly, while elevated WBC, neutrophil, and CRP levels are associated with complicated AA, normal values do not exclude the possibility of both complicated and uncomplicated AA. This is added to the fact that other causes of RLQ abdominal pain may also present with elevated CRP levels, and therefore, CRP alone is not sufficient for diagnosing AA or complicated appendicitis. About 7% of histologically proven pediatric AA cases have normal WBC and CRP levels, unlike in adults [32]. Given the age-dependent nature of some serum inflammatory markers and differences in acute-phase reactions between children and adults [33], diagnosing AA in children remains challenging [34]. We hope our study's findings will contribute to more accurate diagnoses and improved outcomes for children presenting with complicated AA by using more evidence-based findings in the area of biomarkers research.

## 5. Limitations, Strengths, and Future Directions

This study had several limitations. The most prominent was the limited sample size, which may affect the reliability and generalizability of the results. Additionally, the absence of a control group of healthy children hindered our ability to compare parameters between complicated and uncomplicated cases robustly. Another limitation was the restricted number of clinical and laboratory parameters included in the study, which might have constrained the scope of our findings. Moreover, the study's single-center design may limit the applicability of the results to other settings.

Despite these limitations, the study had several strengths. It is one of the first investigations from Iran focusing on complicated AA in children, providing valuable insights into this region's pediatric population. The study employed a robust methodology and rigorous statistical analysis, ensuring that the findings are evidence-based and reliable. Additionally, the identification of CRP, WBC, and neutrophil count as predictive markers for complicated AA offers practical clinical applications for early diagnosis and management.

Future research should be aimed at addressing these limitations by including larger, multicenter cohorts to enhance the generalizability of the findings. Incorporating a control group of healthy children and expanding the range of clinical and laboratory parameters would provide a more comprehensive understanding of the risk factors associated with complicated AA. Developing predictive models and including diverse populations, particularly in developing countries with limited healthcare resources, can further advance the field and improve patient outcomes. Such efforts are crucial for refining diagnostic tools and ensuring timely and effective treatment for pediatric AA worldwide.

## 6. Conclusions

Our study identified WBC count, neutrophil count, and CRP as significant independent predictors of complicated pediatric AA. These laboratory factors, along with their proposed cutoffs, can be effectively used in clinical practice to diagnose and manage children suspected of having complicated AA, potentially improving surgical outcomes in these patients. Future studies with larger sample sizes and more comprehensive factors are recommended to further advance research in this area, especially in developing countries with limited healthcare resources.

## Figures and Tables

**Table 1:** Statistics of demographic, clinical, and laboratory findings of the included sample with acute appendicitis in this study.

**Variable**	**Overall** **N** = 98^**a**^	**Study group**		**p** ** value** ^**b**^
		**Complicated** **N** = 18^**a**^	**Uncomplicated** **N** = 80^**a**^	
Age (y)	9.0 (7.0, 11.0)	9.0 (8.0, 11.0)	9.0 (7.0, 10.5)	0.608
Sex				0.585
Male	60 (61.2%)	10 (55.6%)	50 (62.5%)	
Female	38 (38.8%)	8 (44.4%)	30 (37.5%)	
Evidence of acute appendicitis in US				0.683
Negative	10 (10.2%)	1 (5.6%)	9 (11.3%)	
Positive	88 (89.8%)	17 (94.4%)	71 (88.8%)	
Appendix diameter (cm)^c^	0.8 (0.5, 1.0)	1.0 (0.7, 1.0)	0.7 (0.5, 1.0)	0.076
Duration of symptoms				0.734
≤ 12 h	24 (24.5%)	3 (16.7%)	21 (26.3%)	
24 h	33 (33.7%)	8 (44.4%)	25 (31.3%)	
2–3 days	23 (23.5%)	4 (22.2%)	19 (23.8%)	
4–6 days	13 (13.3%)	3 (16.7%)	10 (12.5%)	
≥ 7 days	5 (5.1%)	0 (0.0%)	5 (6.3%)	
Diarrhea				0.485
Negative	82 (83.7%)	14 (77.8%)	68 (85.0%)	
Positive	16 (16.3%)	4 (22.2%)	12 (15.0%)	
Tenderness				0.305
RLQ	39 (39.8%)	6 (33.3%)	33 (41.3%)	
RLQ and rebound	18 (18.4%)	4 (22.2%)	14 (17.5%)	
RLQ and hypogastric	6 (6.1%)	1 (5.6%)	5 (6.3%)	
RLQ and periumbilical	3 (3.1%)	0 (0.0%)	3 (3.8%)	
RLQ and epigastric	2 (2.0%)	1 (5.6%)	1 (1.3%)	
RLQ and LLQ	2 (2.0%)	1 (5.6%)	1 (1.3%)	
LLQ	2 (2.0%)	0 (0.0%)	2 (2.5%)	
Generalized	8 (8.2%)	2 (11.1%)	6 (7.5%)	
Hypogastric	3 (3.1%)	2 (11.1%)	1 (1.3%)	
Other	7 (7.1%)	1 (5.6%)	6 (7.5%)	
None	8 (8.2%)	0 (0.0%)	8 (10.0%)	
Temperature (°C)	37.0 (36.8, 37.5)	37.0 (36.9, 37.5)	37.0 (36.8, 37.5)	0.864
ESR (mm/h)	17.0 (9.0, 27.0)	23.0 (14.0, 51.0)	15.0 (8.0, 26.0)	0.075
CRP (mg/dL)	11.0 (3.0, 43.0)	34.0 (8.0, 58.0)	8.0 (2.0, 35.0)	0.014
WBC count (⁣^∗^10^9^/*L*)	12.7 (10.2, 16.8)	16.7 (11.6, 21.2)	12.6 (9.9, 15.8)	0.048
WBC count category				0.348
High (≥ 10,000)	76 (77.6%)	16 (88.9%)	60 (75.0%)	
Low (< 10,000)	22 (22.4%)	2 (11.1%)	20 (25.0%)	
Neutrophil count (∗10^9^/*L*)	10.3 (6.7, 14.4)	14.4 (9.6, 17.3)	9.5 (6.1, 13.4)	0.018
Neutrophil count category				0.047
High (≥ 7500)	68 (69.4%)	16 (88.9%)	52 (65.0%)	
Low (< 7500)	30 (30.6%)	2 (11.1%)	28 (35.0%)	
Neutrophil percentage	78.5 (65.1, 84.5)	80.7 (78.3, 86.2)	77.7 (62.2, 84.4)	0.06

Abbreviations: CRP, C-reactive protein; ESR, erythrocyte sedimentation rate; LLQ, left lower quadrant; RLQ, right lower quadrant; US, ultrasound; WBC, white blood cell.

^a^Median (interquartile range) or frequency (%).

^b^Wilcoxon rank sum test; Pearson's chi-squared test; Fisher's exact test.

^c^Excluding the patients with a normal appendix diameter.

**Table 2:** Unadjusted univariable and adjusted multivariable logistic regression analysis of laboratory factors predictive of complicated acute appendicitis in children.

**Biomarker**	**Unadjusted analysis**		**Adjusted analysis** ^**a**^	
	**Odds ratio (95% CI)**	***p* value**	**Odds ratio (95% CI)**	**p** ** value**
ESR	1.03 (1.00–1.05)	**0.037**	1.10 (1.04–1.18)	**0.003**
CRP	1.00 (1.00–1.01)	0.249	1.02 (1.00–1.04)	**0.026**
WBC	1.10 (1.00–1.21)	**0.054**	1.18 (1.03–1.37)	**0.02**
Neutrophil	1.12 (1.01–1.24)	**0.029**	1.23 (1.06–1.45)	**0.009**

*Note:* Statistically significant *p* values (< 0.05) are presented in bold.

Abbreviations: CI, confidence interval; CRP, C-reactive protein; ESR, erythrocyte sedimentation rate; WBC, white blood cell.

^a^Adjusted for age, sex, symptom duration, diarrhea, tenderness, body temperature, and positive evidence of acute appendicitis in ultrasound.

## Data Availability

The data that support the findings of this study are available on request from the corresponding authors. The data are not publicly available due to privacy or ethical restrictions.
